# Multilingual Prediction of Cognitive Impairment with Large Language Models and Speech Analysis

**DOI:** 10.3390/brainsci14121292

**Published:** 2024-12-22

**Authors:** Felix Agbavor, Hualou Liang

**Affiliations:** School of Biomedical Engineering, Science and Health Systems, Drexel University, Philadelphia, PA 19104, USA; fa424@drexel.edu

**Keywords:** large language model, mild cognitive impairment, multilingual processing, speech analysis, whisper, dementia detection

## Abstract

Background: Cognitive impairment poses a significant global health challenge, emphasizing the critical need for early detection and intervention. Traditional diagnostics like neuroimaging and clinical evaluations are often subjective, costly, and inaccessible, especially in resource-poor settings. Previous research has focused on speech analysis primarily conducted using English data, leaving multilingual settings unexplored. Methods: In this study, we present our results from the INTERSPEECH 2024 TAUKADIAL Challenge, where we aimed to automatically detect mild cognitive impairment (MCI) and predict cognitive scores for English and Chinese speakers (169 in total). Our approach leverages Whisper, a speech foundation model, to extract language-agnostic speech embeddings. We then utilize ensemble models to incorporate task-specific information. Results: Our model achieved unweighted average recall of 81.83% in an MCI classification task, and root mean squared error of 1.196 in cognitive score prediction task, which placed the model at the second and the first position, respectively, in the ranking for each task. Comparison between language-agnostic and language-specific models reveals the importance of capturing language-specific nuances for accurate cognitive impairment prediction. Conclusions: This study demonstrates the effectiveness of language-specific ensemble modeling with Whisper embeddings in enabling scalable, non-invasive cognitive health assessments of Alzheimer’s disease, achieving state-of-the-art results in multilingual settings.

## 1. Introduction

Cognitive impairment, encompassing conditions such as Alzheimer’s disease (AD), poses a significant challenge affecting millions of individuals worldwide, without a cure, currently [1]. Early detection and intervention are critical for managing these conditions effectively, yet conventional diagnostic approaches largely rely on subjective assessments and expensive neuroimaging techniques [2].

Currently, the diagnosis of Alzheimer’s disease and related dementias often involves a combination of clinical evaluation, cognitive score testing, such as the Mini Mental State Examination (MMSE) [3], and neuroimaging, etc. In addition to brain scans and cognitive tests, speech analysis has emerged as a promising tool for diagnosing AD. Changes in speech patterns, including disruptions in fluency, vocabulary, and syntactic complexity, have been observed in individuals with cognitive impairment, providing valuable insights into disease progression [4,5,6,7].

The utilization of speech in mild cognitive impairment (MCI) detection offers several advantages. It allows for easy and non-invasive sample collection, streamlining the assessment process. Additionally, the cost-effectiveness and ability to continuously monitor MCI progression make it a valuable tool for large-scale screening. The existing work on utilizing speech for AD detection has primarily been conducted in English [2,8,9,10]. However, the applicability of these findings to multilingual settings, such as English and Chinese, remains unclear.

In recent years, the importance of early detection of cognitive impairment has become increasingly recognized, with growing evidence suggesting that early intervention can delay the progression of conditions like Alzheimer’s disease [11,12,13]. This has fueled a surge in research focused on developing cost-effective, scalable diagnostic tools that can be applied across different populations. As neurodegenerative diseases continue to affect aging populations globally, particularly in countries with limited access to advanced neuroimaging facilities, the need for innovative and accessible diagnostic methods is more urgent than ever [14,15]. Speech analysis offers an excellent opportunity to bridge the gap by providing clinicians with a non-invasive, efficient means for detecting cognitive decline at its early stages. Advances in Artificial Intelligence (AI), particularly large language models (LLMs), have further accelerated progress in this area. These AI-powered technologies allow the analysis of vast amounts of speech data, thus enabling researchers to uncover subtle patterns in language and communication that may be indicative of cognitive decline. Such innovations are paving the way for the deployment of speech-based screening tools in both clinical and community-based settings, enabling earlier identification and subsequent interventions for at-risk populations.

LLMs, such as Whisper [16], have become indispensable tools for speech analysis. Whisper, trained on a massive dataset of multilingual and multitask data, is particularly powerful as it can accurately transcribe speech in various languages and accents, even in noisy environments. Previous approaches [17,18] have used LLMs such as data2vec [19] and wav2vec2 [20] for acoustic analysis of speech data. Other studies [9,21,22] have utilized linguistic features from LLMs such as Bidirectional Encoder Representations from Transformers (BERT) [23] and Generative Pre-trained Transformer 3 (GPT-3) [24] for dementia detection. However, these studies are conducted using only English speech data. In the current study, we use data from the INTERSPEECH 2024 TAUKADIAL Challenge, which consists of speech samples and clinical information for speakers of Mandarin Chinese and English with different levels of cognitive impairment as well as individuals with normal cognition (NC) [25]. Given the global rise in dementia cases [26], the ability to conduct multilingual cognitive assessment has become increasingly important in healthcare.

In this work, we focus on spontaneous speech analysis for automatically detecting people with mild cognitive impairment and predicting a cognitive score on English and Chinese speakers. We explore two primary tasks:

- MCI Classification: This is a classification task, with a goal of distinguishing healthy control speech from mild cognitive impairment (MCI) speech.
- MMSE Score Prediction: This is a regression task, with a goal to infer the subject’s Mini Mental Status Examination (MMSE) scores based on spontaneous speech data.

Our main contributions are as follows. First, we leverage Whisper, a speech foundation model, to extract language-agnostic speech embeddings. Second, we propose a novel ensemble model by incorporating task-specific information, with improvement over competition baseline results [25]. Third, we demonstrate the superiority of language-specific pipelines over language-agnostic methods. Lastly, we evaluate the effectiveness of within-language prediction for detecting MCI as compared to between-language prediction. The integration of majority voting in the ensemble model and task-specific information in our approach represents an innovative strategy to enhance model robustness and accuracy. Majority voting helps to mitigate the risks of overfitting and biases that could arise from individual tasks, ensuring that the final predictions are well-rounded and reflective of the broader cognitive patterns across different task types.

## 2. Materials and Methods

### 2.1. Dataset Description

The dataset used in this work was provided by the organizers of the INTERSPEECH 2024 TAUKADIAL Challenge [23], which consists of Chinese and English speech samples collected while the speakers participated in picture description tasks conducted as cognitive assessments in clinical settings. In the English language dataset, each participant was asked to describe three different pictures: Cookie Theft, Cat Rescue, and Coming and Going picture description tasks. In the Chinese language dataset, participants similarly were asked to provide a detailed description of three pictures depicting Taiwanese culture. The full dataset (English and Chinese) was balanced across age and gender groups to mitigate biases in modeling. It is important to note that the pictures used for descriptions in each language were different.

The training dataset comprises 387 voice recordings from 129 participants from MCI patients and normal cognition (NC) subjects. The test dataset consists of 120 recordings from 40 participants. Within the training set, 222 samples belong to the MCI group, while the remaining 165 samples are healthy control. The dataset is curated to ensure a balanced representation of age and sex among participants, thereby mitigating potential confounding factors and bias using a propensity score matching approach [25,26]. Table 1 shows the basic data description of the participants for age, sex and MMSE scores in the training dataset.

### 2.2. Extracting Linguistic and Acoustic Features from Speech

Acoustic feature extraction is a critical step in analyzing spontaneous speech for cognitive impairment prediction [27,28]. In this study, we employ the Whisper model to extract embeddings representing acoustic features from both Chinese and English datasets.

Whisper is a simple, yet effective, end-to-end approach implemented as an encoder–decoder model. It was trained on a massive dataset of multilingual and multitask supervised data collected from various sources on the web [16]. The extensive training data allows Whisper to effectively capture a wide range of speech patterns and nuances across different languages, resulting in robust acoustic feature extraction for predicting cognitive impairment. In this study, we utilize the *whisper-large-v3* model for acoustic feature extraction. This version of the Whisper model offers embeddings of 1280 dimensions, providing a rich representation of the input speech signals. Notably, the *whisper-large-v3* model represents the latest open-source iteration of the Whisper model available at the time of writing.

The encoder component of the Whisper model generates embeddings, which serve as representations of the acoustic features extracted from the input speech. These embeddings encapsulate relevant acoustic information, facilitating subsequent analysis and prediction tasks.

To represent linguistic features, the speech data underwent transcriptions, respectively, to convert English audio into English text and Chinese audio into Chinese text using Whisper. This step ensures that the linguistic nuances in both English and Chinese speech samples were accurately captured. Following transcriptions, distinct LLMs were applied to obtain text embeddings for each language. For English linguistic features, the *Voyage-large-v2 model* was utilized, which generated text embeddings capable of capturing linguistic information within the English speech data. Similarly, for Chinese linguistic feature representation, the FLAG embedding model, specifically the *FLAG-bge-large-zh-v1.5* variant tailored for Chinese language processing, was employed. We chose these models largely due to their top performance in the Massive Text Embedding Benchmark (MTEB) Leaderboard. Comparisons of linguistic features with other multilingual embedding models did not yield improved performance. Therefore, the results reported in this work are based only on acoustic features.

### 2.3. Model Pipelines

#### 2.3.1. Language-Agnostic Modeling

**Figure 1:**
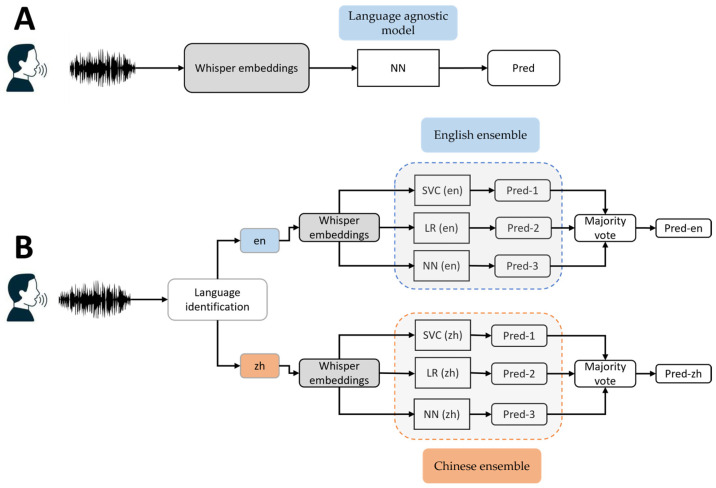
Diagram of language-agnostic (**A**) and language-specific ensemble (**B**) model pipelines.

We started with a language-agnostic model which serves as a baseline for our analysis. This model, depicted in Figure 1A, was designed to be independent of specific languages and aimed to capture general patterns present in the speech data. The baseline model involved extracting embeddings directly from the encoder of the Whisper. These embeddings were further normalized by using z-score normalization, which were then utilized as inputs for classifiers, such as neural networks (NNs). Note that this approach does not incorporate any language identification mechanisms, highlighting the language-agnostic nature of the pipeline.

#### 2.3.2. Language-Specific Ensemble Modeling

For language-specific ensemble modeling, we introduced our approach, as depicted in Figure 1B. This method aimed to leverage the information encoded in three different picture description tasks using an ensemble model. The process began with identifying the language of the speech data, followed by feature extraction using the Whisper model. This step ensures that subsequent modeling processes were tailored to specific language.

For each language, we implemented an ensemble model using a modified stacking technique. This involved training three different machine learning models, each using two of the three picture-description tasks for training and the third task for testing. This setup resulted in three distinct predictions for each subject, based on different tasks. To determine the final prediction, we applied a majority voting strategy, where the most frequent prediction across the three models was selected. This approach leveraged complementary information from the different tasks, ensuring a more robust prediction. Finally, we manually checked the predictions to ensure consistency across the tasks for each subject before submission to the competition organizer.

### 2.4. Experiments

#### 2.4.1. MCI Classification Task

The first task focused on MCI classification, aimed at detecting the presence or absence of MCI based on spontaneous speech recordings. We utilized three machine learning models for this task: a Support Vector Classifier (SVC), Logistic Regression (LR) and neural network (NN). We selected the SVC, LR, and NN, given their proven performance and widespread use in machine learning, as evidenced by our previous research [9,17]. Specifically, the SVC was chosen for its ability to handle high-dimensional data and capture nonlinear relationships through kernel functions, aligning with the complex Whisper embedding space. LR was selected for its simplicity, interpretability, and strong performance in binary classification, providing a baseline. The NN was included to model intricate nonlinear relationships and fully leverage the richness of the Whisper embeddings. By combining these models in an ensemble approach and applying majority voting, we improved the robustness and accuracy of our predictions.

For the classification task, we used two approaches: a language-agnostic model and language-specific model. For language agnostic modeling, we directly used the embeddings from Whisper as input into a neural network, regardless of language. This approach was used as a baseline to compare with the language-specific model. In a language-specific modeling approach, for each language identified and its Whisper embedding, we built an ensemble model where we trained three different machine learning models. Specifically, we trained LR, the SVC, and the NN independently, and combined their predictions using majority voting for a final prediction such that each subject, regardless of the picture description task, should have the same prediction. We additionally implemented the conventional stacking approach using the NN and SVC as the base estimators and LR as the final estimator to compare our task-specific ensemble to the conventional stacking method.

#### 2.4.2. MMSE Score Prediction Task

This is a regression task aimed at predicting MMSE scores based on spontaneous speech recordings. We leveraged the framework developed for the MCI classification for this prediction. In this task, our objective is to predict MMSE cognitive scores using regression models such as Ridge Regression and a neural network. The MMSE scores normally range from 0 to 30, with scores of 26 or higher being considered normal [3]. A score of 20 to 24 suggests mild dementia, 13 to 20 suggests moderate dementia, and less than 12 indicates severe dementia. As such, the prediction is clipped to a range between 0 and 30.

The key difference from the MCI classification lies in the use of regression models tailored for predicting continuous MMSE scores, rather than classification models used for detecting the presence or absence of MCI. As such, instead of using majority voting in classification, we use the average of the predicted MMSE scores.

#### 2.4.3. Between-Language vs. Within-Language Analysis

So far, our ensemble approach has focused on within-language analysis, wherein inferences are performed for tasks with the same language, either English or Chinese. Here, we seek to explore between-language analysis, where we aim to evaluate to what extent transfer learning between languages can be used for MCI detection. Briefly, we first train a machine learning model on the English (Chinese) dataset. Then, we make inferences for a picture description task on the Chinese (English) dataset. To permit direct comparison with the within-language analysis, we use two picture description tasks in the same language (e.g., English) to train the model, and test it with a task in different language (e.g., Chinese). This process allows us to assess the effectiveness of transferring knowledge and model performance across languages.

### 2.5. Performance Evaluation

For the MCI classification task, we assess model performance using the following metrics from the challenge: unweighted average recall (UAR, also called balanced accuracy), specificity, sensitivity, and F1 Score. For the MMSE prediction task, our evaluation metrics include Root Mean Square Error (RMSE) score and Pearson correlation coefficient (i.e., R-squared).

To determine optimal hyperparameters for our models, we adopt a methodology that involves dividing the training data into an 80/20 split for training and validation sets. This split ensures adequate data for both training and validation of the models. Additionally, we use a stratified approach based on both the detected language and diagnosis when dividing the data, ensuring a balanced distribution of classes in the training and validation sets. Such an approach facilitates the effective tuning of hyperparameters and the robust evaluation of model performance, hence enhancing the reliability and generalizability of our findings on the unseen test set.

For the SVC, we tune the regularization parameter and perform a search across various kernel functions (radial basis function, sigmoid function and linear function). The NN model is a feedforward neural network with one hidden layer of size 100. The Adam optimizer is used with a learning rate of 0.001.

## 3. Results

### 3.1. MCI Classification Task

The results of within-language analysis for the English dataset are presented in Table 2, showing the performance metrics of our ensemble approach obtained for inference on each picture description task after model trained on the other two tasks. The results indicate that, in general, majority voting performs better across all the metrics.

Table 3 presents the within-language results for the Chinese dataset, depicting the performance metrics obtained for inferences on each picture description task after the model was trained on the other two tasks. The findings indicate that, overall, majority voting utilizing task-specific information outperforms inferences made using individual picture description tasks.

**Figure 2:**
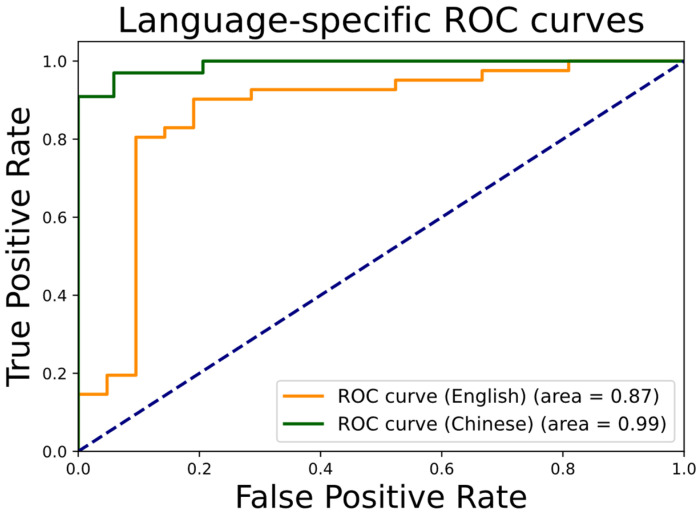
ROC curves of our language-specific model for Chinese and English datasets.

In Figure 2, the receiver operating characteristic (ROC) curves for both the English and Chinese datasets are presented to demonstrate the model classification performance. The area under the ROC curve (AUC) values of 0.87 and 0.99 are achieved, respectively, for English and Chinese datasets. The near-perfect AUC for the Chinese dataset suggests that the model is particularly well-suited to the linguistic and acoustic characteristics of the Chinese language, achieving high accuracy in distinguishing cognitive impairment. These ROC curves provide a clear indication of the model’s robust classification capabilities.

We compare the results of our task-specific approach to the conventional stacking method in Table 4 for both the English and Chinese dataset. It can be seen that for the English data, our task-specific approach achieves a higher balanced accuracy and specificity while having comparable sensitivity and F1 scores. For the Chinese dataset results, our task-specific approach significantly outperforms the conventional stacking method, showing a clear advantage over the conventional method.

Table 5 presents the between-language results obtained from training on the English dataset and inferring on different tasks of the Chinese dataset. The results indicate difficulties in transferring task information from English to Chinese. These results indicate that, despite utilizing task-specific information from the English dataset, the performance of the transfer learning approach in inferring on the Chinese dataset is subpar compared to the within-language results. Similar findings are observed for transfer learning from Chinese to English.

In Table 6, we show the results of the MCI classification task on the unseen test set. Specifically, we compare the performance of the language-agnostic model with the language-specific ensemble modeling approach (Method 2). Results on the unseen test set reveal that the ensemble approach outperforms the language-agnostic model (Method 1) across all the metrics. The language-specific ensemble modeling approach has much better performance in accurately detecting the presence or absence of MCI, with a balanced accuracy of 0.818 compared to 0.612 for language-agnostic methods.

### 3.2. MMSE Score Prediction Task

We present the results of the MMSE cognitive score prediction task in Table 7, where we compare the performance of the language-agnostic model with the language-specific ensemble model. The MMSE prediction results are reported in terms of RMSE scores (lower is better) and the R-squared method (higher is better). These results indicate that the language-specific ensemble modeling approach outperforms the language-agnostic model in terms of both RMSE scores and R-squared.

### 3.3. Comparison with Other Competition Teams

**Figure 3:**
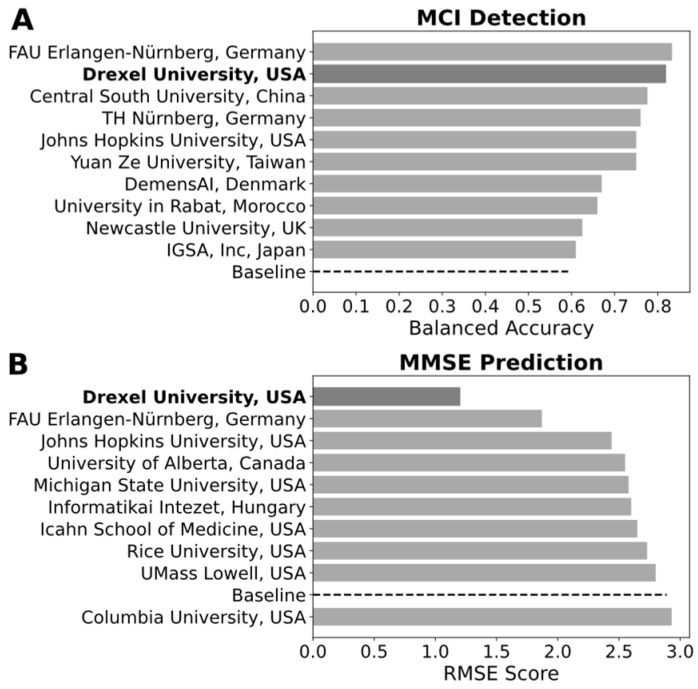
Rankings of the top ten teams and the baseline method in the challenge, assessed using balanced accuracy for the MCI classification task (**A**) where our model ranked second (dark gray), and the RMSE for the MMSE cognitive score prediction task (**B**) where our model ranked first (dark gray). Note that the baseline is represented by black dashed lines.

We compare the performance of our approach with the other participants in the challenge. The competition rankings were based on UAR (i.e., balanced accuracy) for the MCI classification task and root mean squared error (RMSE) for the MMSE cognitive score prediction task. Figure 3 presents the competition results where only the top ten teams are provided for each task, with the baseline also shown. Refer to the full list of results here: (https://taukadial-luzs-69e3bf4b9878b99a6f03aea43776344580b77b9fe54725f4.gitlab.io/ranking.html, accessed on 29 September 2024). We can see from Figure 3 that our model performs remarkably well. We ranked second for MCI detection (Figure 3A), and first for MMSE prediction (Figure 3B).

## 4. Discussion

In this paper, we present the findings of our participation in the INTERSPEECH 2024 TAUKADIAL Challenge, which focused on the automated detection of mild cognitive impairment and the prediction of cognitive scores for both English and Chinese speakers. Our approach harnesses the power of Whisper, a state-of-the-art speech foundation model, to extract language-independent speech embeddings. These embeddings are then integrated into ensemble models to capture task-specific information. Our model achieved an unweighted average recall of 81.83% and a root mean squared error of 1.196 on the MCI classification and cognitive score prediction tasks, respectively. This performance placed our model in the second and first positions in the overall rankings for the respective tasks. Our method relies only on acoustic features extracted from spontaneous speech recordings using Whisper encoder embeddings [14], demonstrating the potential in identifying cognitive impairment based purely on speech. These results highlight the growing potential of speech-based biomarkers in clinical applications, as they offer a non-invasive, scalable, and cost-effective solution for the early detection of cognitive decline.

We performed a comparative analysis of the language-agnostic and language-specific ensemble modeling techniques. Our findings showed a clear advantage of language-specific models in both the MCI classification and MMSE prediction tasks. These models are able to leverage subtle but important language-specific patterns, which play a crucial role in the accurate prediction of cognitive impairment. We additionally utilized task-specific information from the language-specific models by training models on two out of three picture description tasks and testing them on the remaining third task. This process was repeated across all possible combinations of the picture tasks, resulting in three predictions for each language. The final ensemble prediction was obtained through majority voting. Majority voting, a simple yet powerful ensemble technique, allowed us to aggregate three predictions and make a final decision for the same subject in the classification task. In doing so, we ensured that the final prediction was not biased toward any single task but rather reflected the consensus across all task-specific models.

We also conducted an analysis of cross-language predictions, where models trained on data in one language were evaluated on speech data from a different language. This analysis provided insights into the limitations of between-language prediction. We observed a significant reduction in performance when models were applied to different languages from that they were trained on. This drop in accuracy and MMSE prediction underscores the challenge of building generalized models that work across various languages without specific adaptations. This suggests that incorporating language-specific information, even across similar languages, can lead to better predictions than models trained on diverse language datasets without any language-specific adaptations. Our findings highlight the importance of tailoring models to specific linguistic and cultural contexts, especially when dealing with cognitive impairment, where subtle differences in speech can carry diagnostic significance.

MMSE prediction is an important task in clinical settings, as the MMSE is a widely used metric to assess cognitive function. Our results further validate the efficacy of language-specific ensemble modeling techniques for MMSE prediction tasks. In addition to yielding a lower and better RMSE score for the prediction, the use of these models resulted in a higher Pearson correlation coefficient, indicating their effectiveness in accurately predicting MMSE scores from acoustic features. The ability to accurately predict MMSE scores from speech recordings provides a non-invasive and accessible method for monitoring cognitive health, particularly in large-scale screening programs. Moreover, our results indicate that language-specific models are better suited to capturing the relationship between speech patterns and cognitive function. This is consistent with the idea that certain cognitive impairments can manifest differently across languages due to variations in grammar, phonetics, and syntax. By leveraging language-specific features, our ensemble models are able to effectively capture these variations, resulting in accurate MMSE predictions.

One strength of our approach is its reliance solely on acoustic features derived from spontaneous speech. By focusing on acoustic data, we avoid the need for transcribed or labeled text, which can be resource-intensive and time-consuming to obtain, particularly in a clinical setting. Additionally, this approach demonstrates the potential of using spontaneous speech recordings to detect cognitive impairment in a non-invasive and accessible way, which can have far-reaching applications in telemedicine and large-scale screening programs. However, there are some limitations to our study that must be acknowledged. First, while our language-specific models outperformed language-agnostic approaches, we only tested in English and Chinese. Hence, the generalizability of our findings to other languages is unclear. Future work should explore the inclusion of a more diverse set of languages to validate the effectiveness of language-specific modeling across a wider range of linguistic contexts. Second, our focus on acoustic features, while effective, may have missed important linguistic or semantic cues that could further enhance prediction accuracy. This motivates our future work, in which we aim to incorporate text embeddings, as performed in our previous work [9], in addition to using acoustic features to capture a more holistic view of speech and cognitive function. Third, one limitation of this study is the reliance on the MMSE as the primary assessment tool. While widely used, the MMSE is a relatively basic screening instrument with notable shortcomings [29,30], such as limited sensitivity to mild cognitive impairment, susceptibility to ceiling and floor effects, and biases related to education and culture. While our results demonstrate the promise of speech-based biomarkers as complementary tools, the limitations of the MMSE underscore the need for future research to integrate more sensitive and inclusive cognitive assessment instruments to ensure robust and equitable evaluations across diverse populations. Fourth, one of the significant challenges in working with LLMs is the inherent black-box nature of their embeddings. While these embeddings are incredibly powerful in capturing semantic and syntactic meaning, they often lack interpretability. This lack of transparency can hinder our ability to understand why specific predictions are made. As LLM technology continues to advance, it is essential to prioritize interpretability.

While our study demonstrates strong technical performance, it is essential to consider how these findings might translate into practical clinical utility. While our current approach leverages speech-based biomarkers to detect cognitive impairment, its integration into clinical settings would require additional validation with more comprehensive neuropsychological assessments. Future work should explore the application of our model alongside well-established cognitive tests, such as the Montreal Cognitive Assessment (MoCA), or other domain-specific cognitive tests, to evaluate its efficacy in detecting subtle cognitive changes that may not be captured by current screening tools like the MMSE. Furthermore, for our approach to be clinically applicable, we must address key questions regarding thresholds for accurate classification, sensitivity, and specificity in real-world scenarios. This includes the determination of optimal cut-off values for the classification of cognitive impairment across diverse populations, ensuring that the approach maintains an appropriate balance between sensitivity and specificity in all settings. Moreover, the integration of speech-based assessments into existing clinical workflows will require careful consideration of factors such as ease of use, clinician training, and the potential for automating the scoring process.

## 5. Conclusions

This paper presents our results from the INTERSPEECH 2024 TAUKADIAL Challenge, focused on automated detection of mild cognitive impairment (MCI) and cognitive score prediction for English and Chinese speakers. Using Whisper, a leading speech foundation model, we extracted language-independent speech embeddings and incorporated them into ensemble models. Our approach achieved an unweighted average recall of 81.83% for MCI classification (second place) and a root mean squared error of 1.196 for cognitive score prediction (first place). Our method demonstrates the potential of speech-based biomarkers for non-invasive, scalable, and cost-effective early detection of cognitive decline.

Looking ahead, several avenues for future research arise from this study. First, we plan to incorporate text embeddings alongside acoustic features to enhance the model’s ability to capture both the acoustic and linguistic dimensions of speech. By integrating text-based features, we can explore how semantic information and language structure contribute to the detection of cognitive impairment. This multimodal approach could potentially lead to even more accurate predictions of both MCI classification and MMSE scores.

Second, a more detailed comparison between acoustic-based and linguistic-based approaches is warranted. This would provide further insights into how these two types of features interact and whether they offer complementary information that could be leveraged in ensemble modeling. A deeper understanding of these relationships could inform the design of more sophisticated models that make use of both acoustic and linguistic cues to predict cognitive decline.

Finally, we aim to investigate the impact of incorporating additional non-verbal features, such as facial expressions or gestures, which may carry important information about cognitive functions. Multimodal models that combine speech with other behavioral cues could provide a richer picture of cognitive health, offering new opportunities for non-invasive cognitive assessment in diverse populations.

## Figures and Tables

**Table 1:** Basic characteristics of participants for age, sex, and mean MMSE scores with their corresponding standard deviation (sd) in the training data.

Age	MCI			NC		
	M	F	MMSE (SD)	M	F	MMSE (SD)
[60, 65)	12	6	28.0 (1.3)	12	3	29.6 (0.8)
[65, 70)	3	42	26.9 (3.1)	9	57	29.2 (0.9)
[70, 75)	24	51	25.6 (3.9)	15	12	28.1 (1.4)
[75, 80)	30	15	25.4 (4.1)	18	9	29.7 (0.5)
[80, 85)	12	21	25.0 (3.3)	6	21	29.0 (1.0)
[85, 90)	6	0	22.5 (6.0)	3	0	27.0 (0.0)
Total	87	135	25.8 (3.7)	63	102	29.1 (1.1)

**Table 2:** Within-language results on English validation set for SVC, LR and NN. Inferences are made on picture description tasks 1, 2 and 3, respectively. Majority: majority voting. Bold indicates best performance for the metric.

	Inference	Balanced Accuracy	Specificity	Sensitivity	F1
**SVC**	1	0.761	0.619	0.902	0.860
	2	0.701	0.524	0.878	0.828
	3	0.663	0.619	0.707	0.744
	Majority	**0.797**	**0.667**	**0.927**	**0.883**
**LR**	1	0.749	0.619	0.878	0.847
	2	0.712	0.619	0.805	0.805
	3	0.722	**0.762**	0.683	0.757
	Majority	**0.761**	0.619	**0.902**	**0.860**
**NN**	1	0.796	0.714	0.878	0.867
	2	0.712	0.571	0.854	0.826
	3	0.735	0.714	0.756	0.795
	Majority	**0.808**	**0.714**	**0.902**	**0.881**

**Table 3:** Within-language results on Chinese validation set for SVC, LR and NN. Inferences are made on picture description tasks 1, 2 and 3, respectively. Majority: majority voting. Bold indicates the best performance for the metric.

	Inference	Balanced Accuracy	Specificity	Sensitivity	F1
**SVC**	1	0.882	0.794	0.970	0.889
	2	0.896	**0.882**	0.910	0.896
	3	0.897	0.824	0.970	0.901
	Majority	**0.911**	0.853	**0.970**	**0.914**
**LR**	1	0.926	0.853	**1.000**	0.930
	2	0.836	0.853	0.818	0.831
	3	0.926	0.912	0.939	0.925
	Majority	**0.955**	**0.941**	0.970	**0.955**
**NN**	1	0.941	0.882	**1.000**	0.943
	2	0.850	0.882	0.818	0.844
	3	0.926	0.882	0.970	0.928
	Majority	**0.955**	**0.941**	0.970	**0.955**

**Table 4:** Comparison of our task-specific ensemble approach (Task-Specific) to the conventional stacking method (Stacking). Bold indicates the best performance for the metric.

Dataset	Method	Balanced Accuracy	Specificity	Sensitivity	F1
**English**	Task-Specific	**0.808**	**0.714**	0.902	0.881
	Stacking	0.787	0.615	**0.960**	**0.888**
**Chinese**	Task-Specific	**0.955**	**0.941**	**0.970**	**0.955**
	Stacking	0.829	0.809	0.850	0.829

**Table 5:** Between-language results from training on English tasks and inference on Chinese picture description tasks 1, 2, and 3 using SVC, LR, and NN.

Model	Dataset	Balanced Accuracy	Specificity	Sensitivity	F1
**SVC**	1	0.491	0.588	0.394	0.433
	2	0.515	0.029	1.000	0.667
	3	0.506	0.588	0.424	0.459
**LR**	1	0.418	0.382	0.454	0.435
	2	0.431	0.529	0.333	0.367
	3	0.521	0.618	0.424	0.467
**NN**	1	0.529	0.088	0.970	0.667
	2	0.529	0.088	0.970	0.667
	3	0.445	0.647	0.242	0.302

**Table 6:** Classification results on unseen test set for balanced accuracy, specificity, sensitivity and F1 score for language-agnostic and language-specific pipelines using ensemble approach.

Method	Balanced Accuracy	Specificity	Sensitivity	F1
Language-agnostic	0.612	0.620	0.612	0.566
Language-specific	0.818	0.684	0.952	0.851

**Table 7:** RMSE and R-squared values of MMSE prediction task on unseen test set for language-agnostic and language-specific ensemble approaches.

Method	RMSE	R_Squared
Language-agnostic	2.582	0.496
Language-specific	1.196	0.920

## Data Availability

All the data are available at https://dementia.talkbank.org, accessed on 29 September 2024.
